# Association between perioperative potentially inappropriate medication exposure levels and postoperative hospital length of stay among chinese older hospitalized patients: a retrospective cohort study

**DOI:** 10.1186/s12877-025-06848-y

**Published:** 2025-12-17

**Authors:** Kai Gu, Yi Yang, Jiajie Li, Yuheng Chen, Yulin Tang

**Affiliations:** 1 Department of Pharmacy, Sir Run Run Hospital, Nanjing Medical University, Nanjing, China; 2 School of International Pharmaceutical Business, China Pharmaceutical University, Nanjing, China; 3 Department of Pharmacy, The First Affiliated Hospital of Nanjing Medical University, Nanjing, China

**Keywords:** Association, Potentially inappropriate medication, Surgical, Perioperative, Exposure level, Change, Length of stay, Short term

## Abstract

**Background:**

Potentially inappropriate medication (PIM) administration in geriatric surgical patients is increasingly prevalent in the perioperative period. The relation between the variation in PIM exposure and other key postoperative geriatric outcomes, such as postoperative hospital length of stay (POLOS), however, has seldom been reported, and current researches mentioning perioperative PIM concentrate on preoperative long-term home medications or postoperative prescriptions that may not be actually taken. We aimed to investigate whether the level and the change of short-term PIM exposure in the immediate perioperative period was associated with prolonged POLOS (pPOLOS).

**Methods:**

We performed a retrospective cohort study of patients ≥ 65 years of age who underwent elective inpatient surgery at a tertiary academic hospital from July 2022 and March 2023. PIMs were defined using the Beers Criteria as suggested by the American Geriatrics Society. Stage-varying exposure variables were used to quantify cumulative PIM exposure levels during the preoperative (Pre-PIMs), intraoperative (Intra-PIMs), and even the entire perioperative stage period (Total-PIMs, represented by summing Pre-PIMs and Intra-PIMs) for each participant. A multivariable logistic model and restricted cubic spline model were applied to explore the association and dose-response relationship of PIM exposure with the risk of pPOLOS in the total population and subgroups.

**Results:**

196 (44.6%) of 439 participants had a prolonged postoperative length of stay. There was PIM exposure in 378 (86.1%) of the current cohort, analgesics were administered most frequently both preoperatively and intraoperatively albeit via quite different mechanisms of action. Total-PIMs demonstrated superior association with pPOLOS compared to isolated exposure measures, exhibiting greater precision (narrower confidence interval) despite a moderate effect size. This combined metric provided significantly better predictive accuracy (area under the curve=0.763; Delong’s test, *P*<0.01) and a visually distinct linear dose-response relationship, establishing Total-PIMs as a robust independent predictor of pPOLOS risk. In subgroup analysis, significant modification effects of Charlson Comorbidity Index on the association of Total-PIMs with pPOLOS risk were observed.

**Conclusion:**

Combined exposure to Pre-PIMs and Intra-PIMs more independently indicates the risk of pPOLOS in older patients than its individual exposure. These findings could help clinicians to be aware of the possible vulnerability of elderly patients under continued preoperative to intraoperative exposure to PIMs, and highlight the potential value of medication optimization and deprescribing PIMs in the immediate perioperative setting.

**Supplementary Information:**

The online version contains supplementary material available at 10.1186/s12877-025-06848-y.

## Introduction

Currently, all developed countries worldwide have transitioned into aging societies, while many developing countries are either in the process of or will soon experience this demographic shift [1]. As a developing country with the fastest aging rate and a large population, China has the largest population of elderly individuals who are aging far before they become wealthy. Furthermore, the proportion of elderly patients undergoing elective surgery is rapidly increasing due to remarkable advances in surgical and anaesthetic techniques, as well as growing demand for elderly healthcare [2]. Age-related changes in pharmacokinetics and pharmacodynamics can significantly modify the response to perioperative medications, leading to an increased susceptibility to the occurrence of adverse drug reactions (ADRs), and a decrease in the ability to adapt to external stressful events such as surgical procedures [3]. Consequently, compared to younger patients, elderly individuals are at much higher risk of experiencing adverse postoperative events and demanding additional resource intensive perioperative care [4]. As hospitals face pressure to reduce health care costs while improving the quality of healthcare resource delivery, the identification of modifiable risk factors in older surgical patients is essential for developing targeted interventions to enhance postoperative outcomes.

Potentially inappropriate medication (PIM) users represent a potentially high-risk surgical population, and perioperative care may need to be tailored. Despite medication avoidance is recommended based on Beers Criteria (a reference tool commonly used for defining PIM use in people ≥ 65 years of age) to prevent adverse drug events such as postoperative delirium, PIM administration in geriatric surgical patients is increasingly prevalent in the perioperative period [5–7]. However, data regarding the impact of PIM use perioperatively on postoperative outcomes in geriatric surgical patients remain relatively sparse and generally limited by focusing on long-term home medications before surgery [8–10] or prescriptions after surgery [11, 12] that may not be actually taken, which may not accurately reflect the effect of short-term medications administered during the immediate perioperative period. Although the short-term inpatient medications are the primary means for preoperative optimization of patient risk factors and intraoperative maintenance of physiological homeostasis, the risks of adverse effects should be balanced against the potential benefits of treatment, even in a surgical population. Awareness and modulation of medication-specific risk factors in the pre- (covering the bridging period) and intra-operative settings are crucial for timely intervention and ultimately improve short-term postoperative outcomes [13]. Noteworthly, however, whether and how increased quantity or degree of PIMs exposure are associated with adverse outcomes in elderly elective surgical patients remains under-studied, especially in China, world's most populous country.

In a recent meta-analysis that pooled data from 63 studies for nonsurgical patients, multiple PIMs exposure (3 and more) are independently associated with a 26% to 73% increase in the risk of adverse drug events, as well as a 11% to 30% increase in the odds of hospital readmissions, emergency department visits and extended LOS [14]. Moreover, a few previous studies conducted on orthopedic patients in North America showed consistent directional associations between rising postoperative PIM exposure levels and increased risks of a longer time to full functional recovery, readmission and emergence department utilization, supporting a dose-response relationship that could underlie a causal link between PIM exposure and short-term adverse postoperative outcomes[11, 12]. Given the overall consistency of PIM exposure-response patterns across studies, we theorized that the variation in PIM exposure across pre- to intra-operative stage periods may significantly affects other key geriatric outcomes after surgeries such as postoperative hospital length of stay (POLOS), which have not been explored in detail. POLOS is an objective and quantifiable indicator that serves as a reliable proxy for evaluating postoperative outcomes, the quality of care, and healthcare costs after elective surgeries[15, 16]. Overall, if this is the case, the use of PIM represents a potentially modifiable risk factor and provides a unique target for surgical quality improvement.

As noted above, it has been hypothesized that geriatric patients undergoing elective surgery exposed to short-term PIMs during the immediate perioperative period would experience an increased rate of delayed discharge, even after adjustment for relevant clinical covariates. Therefore, our specific objective was to measure the adjusted association of changes in PIM exposure with POLOS in a mixed surgical population at a single, urban academic institution.

## Methods

### Study design and patient population

This study is a retrospective, cross-sectional analysis of an administrative database of adults ≥ 65 years of age who underwent elective non-cardiac surgery between July 2022 and March 2023 at Sir Run Run Hospital Affiliated to Nanjing Medical University in China, a tertiary academic hospital with approximately 750 beds. Data extracted from electronic medical records were coded to ensure confidentiality for privacy concerns. We only included the index surgery if multiple surgeries were performed during the study period. Patients who met any of the following criteria were excluded: 1) incomplete peioperative medication data; 2) patients who died in the hospital; and 3) patients with emergency surgeries or those admitted to the ICU ahead of surgery. As the primary interest of this study was the impact of perioperative PIM exposure on postoperative outcomes, exclusion criteria were predefined prior to any analysis.

### Covariates: baseline characteristics

Demographic details and perioperative information obtained from the electronic medical records were reviewed to describe the diversity of the study population, which included age, sex, body mass index (BMI), Charlson Comorbidity Index (CCI), American Society of Anesthesiologists (ASA) score, site of surgery (intra-thoracic or abdominal, pelvic and peripheral), type of anesthesia, and duration of anesthesia. Presence of comorbidities (including pulmonary circulatory disease, diabetes mellitus, cardiac-cerebral vascular disease, neurodegenerative disease, rheumatic disease, cancer, peptic ulcer, and hypertension) at the time of surgery were selected based on their clinical significance and for inclusion in the Charlson Comorbidity Index, which either have been associated with PIM use or adverse outcomes after surgery [17, 18]. Preoperative biochemical values including creatinine clearance rate (Ccr), albumin (ALB), and hemoglobin (HGB) were also collected and cases with missing albumin(3.4% of total sample) or hemoglobin data (0.2% of total sample) were imputed using multiple imputation with five iterations, which is considered adequate for the proportion of missing data.

Because postoperative complication could influence hospital length of stay (LOS), geriatric researchers trained specifically for this study adjudicated and recorded each postoperative complication by extensively reviewing medical records. Major complications within 30 days following surgery include surgical site infection, acute renal failure, postoperative bleeding requiring transfusion, unplanned intubation, deep venous thrombosis, stroke, myocardial infarction, sepsis, pneumonia, and delirium. A composite binary variable for postoperative morbidity was created to indicate the incidence of one or more complications.

Ultimately, based on biological plausibility and prior empirical evidence for confounding the relationship between PIM administration status and adverse outcomes, all the above-mentioned baseline characteristics were converted into covariates and included in the regression model for risk adjustment.

### Exposure: perioperative potentially inappropriate medication measurement

The perioperative period began on arrival in the preoperative ward and ended at return to the ward following anesthesia, including pre- and intra-operative stage periods. As prior analyses of preoperative medications mainly relied on patient recall, which is notoriously inaccurate, especially in the elderly population, we focused solely on capturing information of short-term medications with systemic efficacy during the hospital stay.

PIM exposure was quantified by merging patient in-hospital medication information with the 2019 AGS Beers Criteria^®^ list, identifying and summing the number of filled entries for all medications to generate individual-level continuous PIM exposure frequencies measured over the span of five Beers categories (divided into Beers 1: medications to avoid in older adults; Beers 2: medications to avoid due to drug-disease or drug-syndrome interactions; Beers 3: medications to use with caution; Beers 4: medications to avoid due to potentially clinically important drug-drug interactions; Beers 5: medications that should be avoid based on varying levels of kidney function in older adults) [19]. Due to variation in PIM exposure frequencies over stage, stage-varying exposure variables were used to estimate cumulative PIM exposure levels during the preoperative (Pre-PIMs), intraoperative (Intra-PIMs), and even the entire perioperative stage period (Total-PIMs, represented by summing Pre-PIMs and Intra-PIMs) for each participant.

Given the exploratory nature of the current research hypothesis, we also conducted an ancillary analysis of categorical indicators for possible combinations of Pre-PIMs and Intra-PIMs. There were three exposure levels, namely high exposure (receipt of both Pre-PIMs and Intra-PIMs), low-moderate exposure (receipt of either only Pre-PIMs or Intra-PIMs), and no exposure (receipt of neither).

### Outcome measures

The outcome of interest was prolonged postoperative hospital length of stay (pPOLOS), which was defined as a postoperative hospital length of stay (number of nights in the hospital from the date of surgery until date of discharge) greater than the observed median LOS (vs ≤ median) after elective non-cardiac surgery in the current cohort. As we all know, POLOS was influenced by factors such as type of surgery and medical insurance policy, but relevant studies had also raised the possibility that when LOS varied widely under the same conditions, the median could guide interpretation of sudden changes in postoperative physiological status associated with treatment adverse events [20]. Therefore, the authors used the median of POLOS as a threshold for whether the postoperative LOS was extended.

### Statistical analysis

Descriptive statistics on study variables were presented as mean with standard deviation, median with interquartile range, or frequency and percentage as deemed appropriate. Normality was assessed using qq plots and histograms. The t-test or one-way analysis of variance for normally distributed continuous variables, Mann-Whitney or Kruskal-Wallis test for non-normal continuous variables, and the chi-squared test or Fisher’s exact test for categorical variables were used to analyze differences in baseline characteristics between groups.

Missing data that were considered missing at random were treated with multiple imputation, with number of iterations dictated by proportion of missing data, using the misc package in the R statistical software (version 4.3.2, http://www.R-project.org/).

Univariate and multivariate logistic regression analysis were performed with factors important for pPOLOS occurrence. Independent factors were assessed univariately and included in the multivariable analyses if the univariable *P*-value was <0.10. We assessed the model covariates that were ultimately included in the analyses for collinearity, removing those with variance inflation factors greater than 4. The estimated odds ratio (OR) with their 95% confidence interval (CI) were calculated using the survey package in R. The crude model (Model 1) did not adjusts any potential confounders. In Model 2, we adjusted for inherent demographic factors including age, sex, and BMI. In Model 3, we further adjusted for CCI, ASA, site of surgery, type of anesthesia, duration of anesthesia, comorbidities, preoperative biochemical values, and postoperative complication morbidity. Separating the influence of cumulative exposure to PIMs preoperatively from intraoperatively could have facilitated a quantitative discussion of the advantages or disadvantages of Pre-PIMs and Intra-PIMs in tandem compared with their application alone, as well as allowing further understanding of how various PIM exposure measures shape clinical decision-making. We therefore embedded a continuous and a categorical model into the multivariable logistic regression model. In the continuous model, we estimated three stage-varying measures of exposure: Pre-PIMs, Intra-PIMs, and Total-PIMs, which summed Pre-PIMs and Intra-PIMs. In the categorical model, the extents of cumulative PIMs exposure perioperatively were categorized into three levels: high exposure, low-moderate exposure, and no exposure, using the method we mentioned above. The discriminatory ability of models was assessed using the area under the receiver operating characteristic curve (AUC-ROC), with AUC≥0.75 indicating adequate classification performance; differences between AUCs were evaluated using DeLong's test. Besides, three restricted cubic spline (RCS) curves with 4 knots located at the 20th, 40th, 60th and 80th percentiles of the exposure distribution were further plotted using the rms package in R to evaluate the shape of the association in the continuous model, and the adjusted confounding factors in RCS were the same as those adjusted in the logistic regression model 3.

Finally, we performed exploratory subgroup-level analyses to examine potential modification effects of various stratifying variables on the association of Total-PIMs with pPOLOS occurrence. Heterogeneity between groups was assessed by likelihood ratio tests comparing the models with and without the multiplicative interaction terms.

All statistical analyses were performed using R version 4.3.2 software. Two-sided *P* < 0.05 was considered statistically significant (* *P*<0.05, ** *P*<0.01, *** *P* < 0.001).

### Ethics approval and consent to participate

This study was conducted in accordance with the Declaration of Helsinki. The study protocol was approved by the Institutional Research and Ethics Committee (IREC) of Sir Run Run Hospital (Reference No: 2022-SR-S034). Then we obtained clearance from Sir Run Run Hospital and informed consent from each participant.

## Results

### Baseline characteristics of study population

**Fig. 1:**
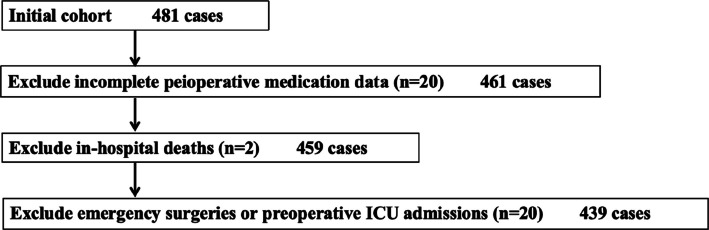
Flowchart of excluded cases

The initial dataset comprised 481 surgical cases. After applying exclusion criteria (Figure 1), 439 patients constituted the final analytic cohort. Table 1 shows the baseline characteristics of these participants stratified according to the study outcome. Median postoperative stay was 6 days, with 196 (44.6%) participants defined as prolonged postoperative length of stay. Among those with prolonged POLOS, half (median of extended POLOS) stayed for more than six extra days (total POLOS 12 days) and 25% of them stayed longer than nine extra days (total POLOS 15 days). Compared with the non-pPOLOS group, participants with pPOLOS tended to be older and frailer; they had a higher prevalence of pulmonary circulatory disease, diabetes, cardiac-cerebral vascular disease, rheumatic disease, cancer and peptic ulcer, and had higher CCI, ASA levels and lower ALB, HGB levels. They were also more likely to undergo intra-thoracic or abdominal surgery under prolonged anaesthesia or general anaesthesia, and to suffered a greater probability of postoperative complications (all *P*<0.05).

**Table 1:** Baseline characteristics of study population according to the presence of prolonged postoperative length of stay

Characteristics	Prolonged postoperative length of stay		*P* value
	No (*N*=243)	Yes (*N*=196)	
Demographics			
Age, years			0.012
<70	118 (48.6)	71 (36.2)	
≥70	125 (51.4)	125 (63.8)	
Sex			0.504
Male	125 (51.4)	108 (55.1)	
Female	118 (48.6)	88 (44.9)	
BMI, kg/m^2^			0.667
<23	103 (42.4)	88 (44.9)	
≥23	140 (57.6)	108 (55.1)	
Comorbidities			
Pulmonary circulatory disease	33 (13.6)	50 (25.5)	0.002
Diabetes mellitus	47 (19.3)	59 (30.1)	0.012
Cardiac-cerebral vascular disease	55 (22.6)	62 (31.6)	0.044
Neurodegenerative disease	16 (6.6)	19 (9.7)	0.308
Rheumatic disease	14 (5.8)	23 (11.7)	0.039
Cancer	45 (18.5)	94 (47.9)	<0.001
Peptic ulcer	13 (5.3)	22 (11.2)	0.037
Hypertension	136 (55.9)	119 (60.7)	0.366
CCI			<0.001
<5	147 (60.5)	52 (26.5)	
≥5	96 (39.5)	144 (73.5)	
ASA			<0.001
<3	197 (81.1)	128 (65.3)	
≥3	46 (18.9)	68 (34.7)	
Site of surgery			0.008
Peripheral	103 (42.4)	58 (29.6)	
Intra-thoracic or abdominal	62 (25.5)	73 (37.2)	
Pelvic	78 (32.1)	65 (33.2)	
Type of anesthesia			<0.001
Spinal or epidural	86 (35.4)	33 (16.8)	
Nerve block	34 (14.0)	6 (3.1)	
General	123 (50.6)	157 (80.1)	
Duration of anesthesia, min			<0.001
<120	161 (66.3)	37 (18.9)	
≥120	82 (33.7)	159 (81.1)	
Preoperative biochemical values			
Creatinine clearance rate, mL/min			1.000
<60	76 (31.3)	61 (31.1)	
≥60	167 (68.7)	135 (68.9)	
Albumin, g/L, median (25th, 75th)	41.0 (37.6, 44.5)	35.2 (31.5, 40.2)	<0.001
Hemoglobin, g/L, median (25th, 75th)	127.0 (113.0, 136.5)	112.5 (96.8, 127.2)	<0.001
Postoperative complication morbidity			<0.001
No complication	234 (96.3)	135 (68.9)	
Incidence of 1 or more complications	9 (3.7)	61 (31.1)	
Total-PIMs, frequencies, median (25th, 75th)	3.0 (1.0, 4.0)	5.0 (4.0, 6.0)	<0.001
Postoperative length of stay, days, median (25th, 75th)	3.0 (2.0, 5.0)	12.0 (8.0, 15.2)	<0.001

All categories are shown as frequency and (percentage) unless otherwise indicated

*Abbreviations*: *BMI* body mass index, *CCI* Charlson Comorbidity Index, *ASA* American Society of Anesthesiology, *PIM* potentially inappropriate medication

### PIM frequency distribution

The cumulative frequencies of exposure perioperatively ranged from 0 to 11, although 99% of cases being equal to or less than 9. The median value of Total-PIMs in the pPOLOS group [5] was significantly higher than that in the non-pPOLOS group [3] (Table 1). As shown in Table 2, Total-PIMs levels were significantly higher in olders and participants with diabetes, cardiac-cerebral vascular disease, neurodegenerative disease, cancer, CCI≥5, ASA≥3, intra-thoracic or abdominal surgery, general anesthesia, duration of anesthesia≥120min or postoperative complications than that in their corresponding reference groups (all *P* < 0.05). Besides, a similar stepwise incremental relationship between different exposure levels and pre-existing medical condition was also observed in the categorical analysis of perioperative cumulative PIMs exposure. There was PIMs exposure in 378 (86.1%) of the current cohort. The prevalence of comorbidities and high disease burden rates (CCI≥5 or ASA≥3) increased with higher level of PIMs exposure in the other two groups, in comparison with participants without PIMs exposure (Supplementary Table).

**Table 2:** Distributions of cumulative frequencies of PIMs exposure perioperatively and odds ratio (95% confidence interval) for pPOLOS in different subgroups

Variables	N (%)	Total-PIMs		pPOLOS	
		median (25th, 75th)	*P* value	OR (95% CI)	*P*_interaction_
Age, years			0.015		0.459
<70	189 (43.1)	3.0 (1.0, 5.0)		1.27 (1.01, 1.63)	
≥70	250 (56.9)	4.0 (2.0, 6.0)		1.23 (1.02, 1.49)	
Sex			0.155		0.819
Male	233 (53.1)	3.0 (1.0, 5.0)		1.21 (1.00, 1.49)	
Female	206 (46.9)	4.0 (3.0, 5.0)		1.23 (0.98, 1.54)	
BMI, kg/m^2^			0.978		0.523
<23	191 (43.5)	4.0 (2.0, 5.0)		1.21 (0.98, 1.52)	
≥23	248 (56.5)	4.0 (2.0, 5.3)		1.20 (0.98, 1.48)	
Pulmonary circulatory disease			0.047		0.102
No	356 (81.1)	3.0 (1.8, 5.0)		1.14 (0.98, 1.33)	
Yes	83 (18.9)	4.0 (3.0, 6.0)		NA	
Diabetes mellitus			<0.001		0.561
No	333 (75.9)	3.0 (1.0, 5.0)		1.19 (1.02, 1.40)	
Yes	106 (24.1)	5.0 (3.0, 6.0)		1.38 (1.00, 1.98)	
Cardiac-cerebral vascular disease			0.013		0.381
No	322 (73.3)	3.0 (1.0, 5.0)		1.24 (1.06, 1.47)	
Yes	117 (26.7)	4.0 (3.0, 5.0)		1.18 (0.84, 1.68)	
Neurodegenerative disease			<0.001		0.171
No	404 (92.0)	3.5 (1.8, 5)		1.18 (1.02, 1.38)	
Yes	35 (8.0)	5.0 (3.5, 6.0)		NA	
Rheumatic disease			0.075		0.140
No	402 (91.6)	4.0 (2.0, 5.0)		1.25 (1.07, 1.46)	
Yes	37 (8.4)	4.0 (3.0, 5.0)		0.86 (0.45, 1.69)	
Cancer			<0.01		0.168
No	300 (68.3)	3.0 (1.0, 5.0)		1.19 (1.01, 1.40)	
Yes	139 (31.7)	5.0 (3.0, 6.0)		1.43 (0.91, 2.27)	
Peptic ulcer			0.991		0.77
No	404 (92.0)	4.0 (2.0, 5.0)		1.22 (1.06, 1.42)	
Yes	35 (8.0)	4.0 (2.5, 5.0)		NA	
Hypertension			0.335		0.801
No	184 (41.9)	3.5 (2.0, 5.0)		1.17 (0.95, 1.45)	
Yes	255 (58.1)	4.0 (2.0, 5.0)		1.21 (0.99, 1.48)	
CCI			<0.01		0.006
<5	199 (45.3)	3.0 (1.0, 4.5)		1.05 (0.85, 1.29)	
≥5	240 (54.7)	4.0 (3.0, 6.0)		1.36 (1.09, 1.71)	
ASA			0.001		0.233
<3	325 (74.0)	3.0 (1.0, 5.0)		1.24 (1.04, 1.49)	
≥3	114 (26.0)	4.0 (3.0, 6.0)		1.33 (0.98, 1.85)	
Site of surgery			0.001		0.484
Peripheral	161 (36.7)	3.0 (2.0, 5.0)		1.19 (0.95, 1.50)	
Intra-thoracic orabdominal	135 (30.8)	4.0 (3.0, 6.0)		1.49 (1.02, 2.33)	
Pelvic	143 (32.6)	3.0 (1.0, 5.0)		1.28 (0.92, 1.79)	
Type of anesthesia			<0.01		0.099
Spinal or epidural	119 (27.1)	1.0 (0.0, 3.0)		1.17 (0.89, 1.55)	
Nerve block	40 (9.1)	2.0 (1.0, 3.3)		4.58 (0.63, 33.10)	
General	280 (63.8)	5.0 (3.0, 6.0)		1.19 (0.99, 1.45)	
Duration of anesthesia, min			<0.01		0.553
<120	198 (45.1)	3.0 (1.0, 4.0)		1.31 (1.04, 1.66)	
≥120	241 (54.9)	5.0 (3.0, 6.0)		1.14 (0.94, 1.40)	
Ccr, mL/min			0.708		0.181
<60	137 (31.2)	3.0 (2.0, 5.0)		1.30 (1.02, 1.70)	
≥60	302 (68.8)	4.0 (2.0, 5.0)		1.17 (0.98, 1.39)	
Presence of postoperative complications			<0.01		0.35
No	369 (84.1)	3.0 (1.0, 5.0)		1.20 (1.03, 1.39)	
Yes	70 (15.9)	5.0 (3.0, 7.0)		1.79 (0.69, 9.05)	

*Abbreviations*: *pPOLOS* prolonged postoperative hospital length of stay, *BMI* body mass index, *CCI* Charlson Comorbidity Index, *ASA* American Society of Anesthesiology, *Ccr* creatinine clearance rate, *PIM* potentially inappropriate medication, *NA* not available. The odds ratio (95% confidence interval) was calculated in the continuous model adjusting age, sex, BMI, CCI, ASA, site of surgery, type of anesthesia, duration of anesthesia, comorbidities, preoperative biochemical values, and postoperative complication morbidity

### PIM Characteristics

**Fig. 2:**
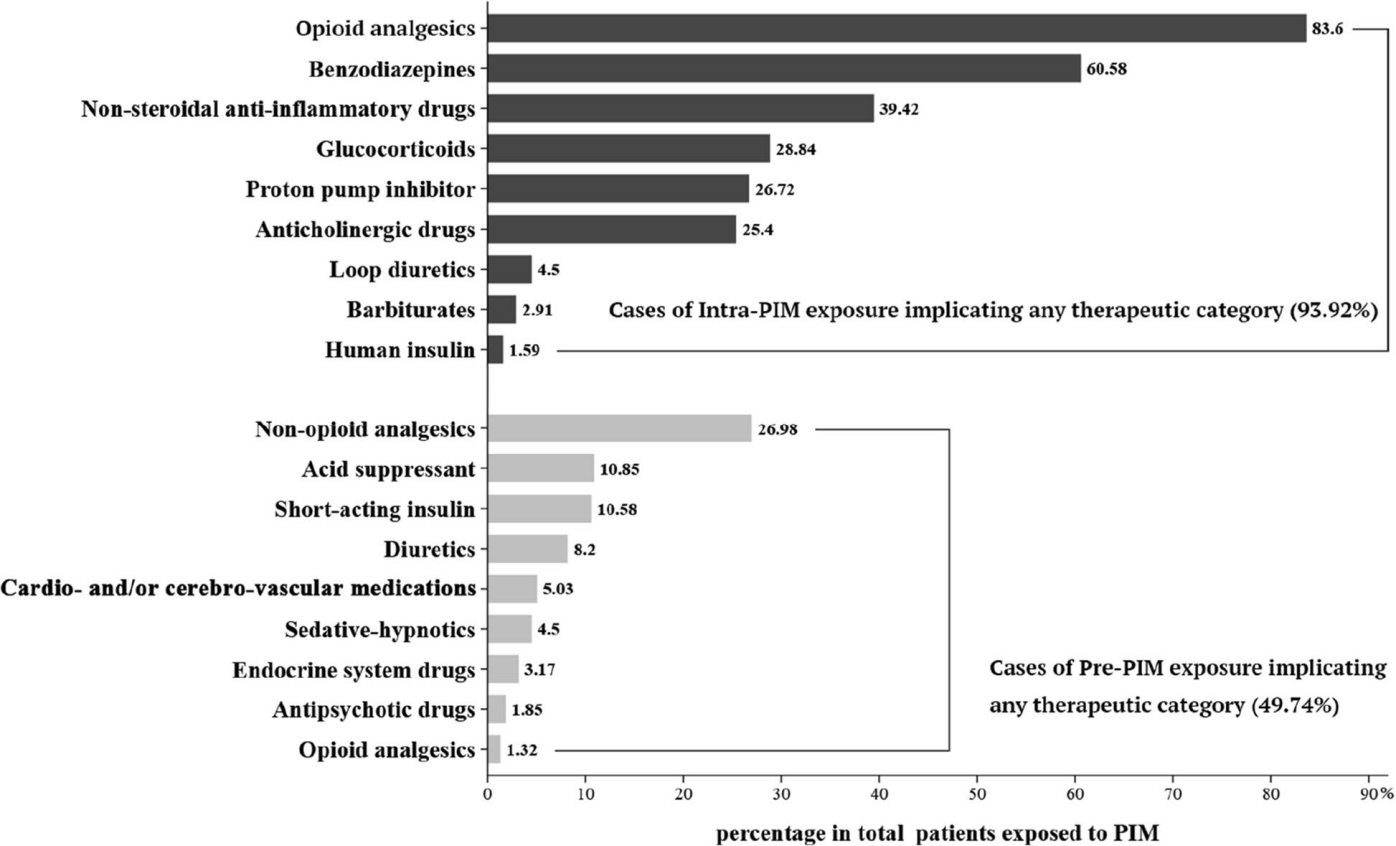
Percentage of cases that implicated each therapeutic category of drugs in total patients exposed to PIM (*n* = 378). Abbreviation: PIM, potentially inappropriate medication

Among older participants with receipt of any PIM across pre- to intra-operative stage periods, 188 (49.7%) and 355 (93.9%) were treated with at least one Pre-PIM and Intra-PIM, respectively. The distribution of Pre-PIMs and Intra-PIMs in different therapeutic categories was summarized in Fig. 2. Analgesics were administered most frequently both preoperatively and intraoperatively albeit via quite different mechanisms of action.

### PIM Exposure and pPOLOS

**Fig. 3:**
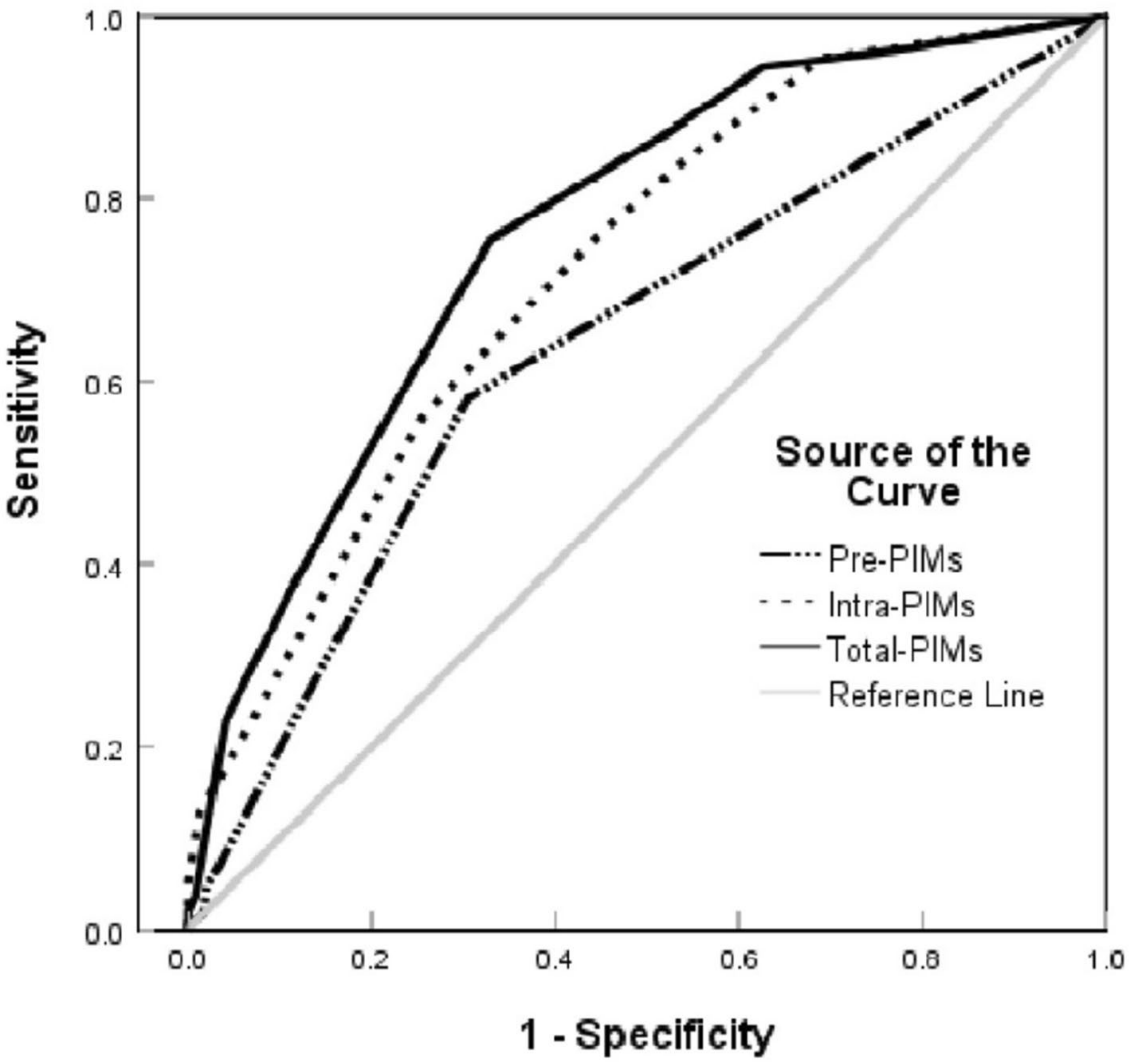
ROC analysis for predicting pPOLOS by Pre-PIMs, Intra-PIMs, and Total-PIMs. AUCPre-PIMs=0.640, AUCIntra-PIMs=0.723, AUCTotal-PIMs=0.763

**Fig. 4:**
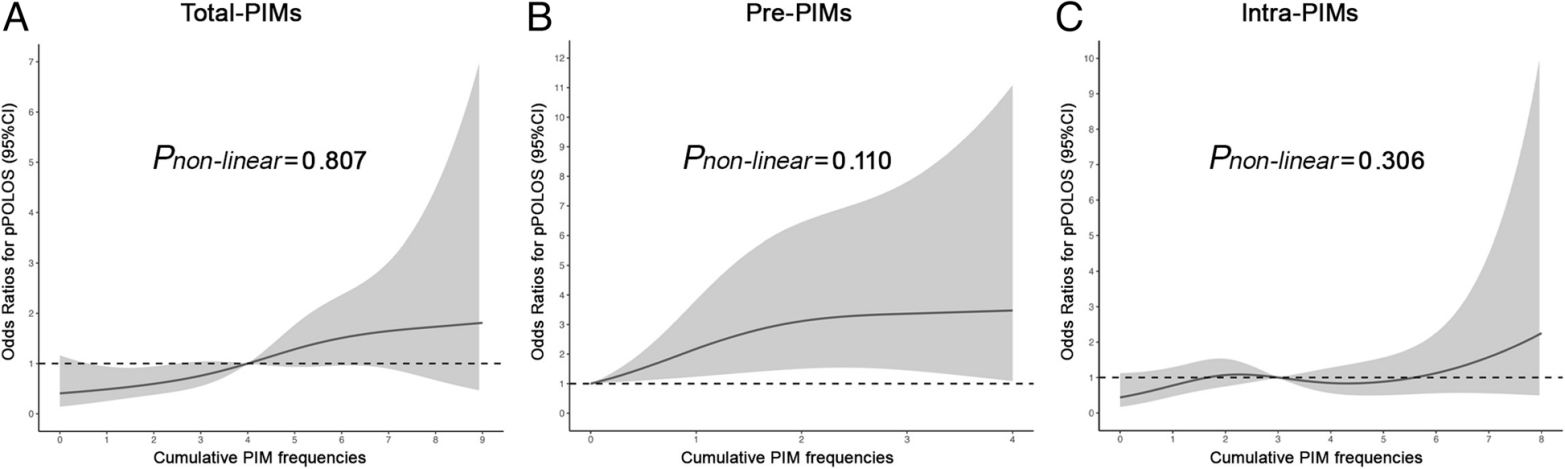
The dose-response relationships of Total-PIMs, Pre-PIMs and Intra-PIMs with the risk of pPOLOS in total population. Abbreviation: PIM, potentially inappropriate medication; pPOLOS, prolonged postoperative hospital length of stay. The solid black lines represented the ORs of pPOLOS, the gray region indicated corresponding 95% CIs. The short dashed black lines indicated the reference value. The *P*-value for non-linear association <0.05 indicated a nonmonotonic dose-response curve

As shown in Table 3, the fully adjusted categorical model revealed that isolated Pre-PIMs or Intra-PIMs exposure (low-moderate level) showed no direct association with pPOLOS occurrence, whereas co-exposure (high level) significantly increased pPOLOS risk (adjusted OR = 3.13, 95% CI: 1.24–8.80.24.80, *P* = 0.021). In the continuous model, both Total-PIMs and Pre-PIMs demonstrated significant associations with increased pPOLOS risk in the overall population, with adjusted ORs of 1.21 (95% CI: 1.06–1.40.06.40, *P* = 0.006) and 1.41 (95% CI: 1.08–1.85.08.85, *P* = 0.01) respectively, suggesting cumulative effects that intensify with higher exposure levels. Notably, despite Total-PIMs exhibiting a moderate effect size (OR 1.21 vs 1.41 vs 1.14), its narrower confidence interval indicates greater precision in effect estimation. (Fig. 3) ROC analysis further confirmed that the Total-PIMs model provided superior predictive performance for pPOLOS (AUC = 0.763), significantly outperforming both Pre-PIMs and Intra-PIMs models (Delong’s test, *P*< 0.01) and meeting the threshold for good classification (AUC≥0.75). Furthermore, RCS modeling indicated monotonic dose-response relationships for all three exposure measures (*P*_non-linear_ for Total-PIMs = 0.807, *P*_non-linear_ for Pre-PIMs = 0.110, *P*_non-linear_ for Intra-PIMs = 0.306), with Total-PIMs demonstrating a visually more pronounced linear dose-response trend than the other exposure metrics (Figure 4). Collectively, these findings suggest greater statistical robustness in the Total-PIMs association and underscore that the combined quantitative assessment of preoperative-to-intraoperative cumulative PIMs exposure provides more independent predictive value for pPOLOS risk than individual exposure measures.

**Table 3:** Logistic regression analysis for the association of PIMs exposure with pPOLOS

Outcomes	Continuous models			Categorical models		
	Pre-PIMs	Intra-PIMs	Total-PIMs	No exposure	Low-moderate exposure	High exposure
Model 1	1.53 (1.27, 1.87)***	1.49 (1.35, 1.65)***	1.57 (1.43, 1.75)***	1.00 (ref)	4.29 (1.97, 10.75)***	16.29 (7.37, 41.41)***
Model 2	1.53 (1.26, 1.87)***	1.49 (1.35, 1.66)***	1.58 (1.43, 1.76)***	1.00 (ref)	4.54 (2.07, 11.46)***	17.44 (7.80, 44.90)***
Model 3	1.41 (1.08, 1.85)*	1.14 (0.98, 1.33)	1.21 (1.06, 1.40)**	1.00 (ref)	1.07 (0.42, 2.99)	3.13 (1.24, 8.80)*

*Abbreviations*: *pPOLOS* prolonged postoperative hospital length of stay, *PIM* potentially inappropriate medication; Model 1, the crude model; Model 2, adjusted for inherent demographic factors including age, sex, and BMI; Model 3, further adjusted for CCI, ASA, site of surgery, anesthetic technique, anesthetic operative time, comorbidities, preoperative biochemical values, and postoperative complication. *P<0.05, **P<0.01, ***P< 0.001

### Subgroup analyses

In exploratory subgroup analyses (Table 2), we observed no statistically significant heterogeneity in prolonged postoperative hospitalization risk associated with increased Total-PIMs exposure across subgroups (all interaction *P* values >0.05), except among the subgroup of older participants at higher burden of comorbidities (*P*_interaction_ = 0.006). Total-PIMs was significantly associated with a 1.36-fold (95%CI: 1.09, 1.71) adjusted increased risk of pPOLOS in CCI ≥ 5 groups.

## Discussion

China's transition to bundled payment systems heightens the need for perioperative risk stratification in elderly surgical patients. Building on evidence that medication-based models predict cost-related outcomes like prolonged LOS [21, 22], this study addresses a critical gap regarding adverse effects of dynamic PIM exposure. Our findings demonstrate that increasing short-term PIM exposure in Chinese elderly non-cardiac surgery patients exhibits a stepwise association with prolonged hospitalization risk. Crucially, combined preoperative-intraoperative PIMs exposure confers significantly greater risk than phase-specific exposure alone.

### Clinical burden and cumulative effects of PIMs exposure

Elderly surgical inpatients face elevated risks of both PIM prescription and adverse outcomes from such exposure. Despite growing risk awareness, perioperative PIMs use has increased temporally. While prior studies reported 69% PIMs prevalence among frail surgical patients [23], our cohort revealed higher exposure in Chinese elderly surgical patients (76–92%), aligning with non-surgical populations [24]. This reflects delayed adoption of PIM concepts in China and suboptimal adherence to Beers Criteria [25]. Barriers to perioperative PIMs identification include management uncertainties for screen-positive patients and limited epidemiology of short-term exposure. To our knowledge, this is the first cohort study analyzing dynamic preoperative-to-intraoperative PIMs exposure against postoperative outcomes. Our models consistently demonstrated dose-response relationships for prolonged hospitalization risk, corroborating prior arthroplasty studies [12] and nonsurgical population data linking PIMs use with functional decline, extended stays, readmission, and mortality [26–29]. Collectively, we strengthen evidence for short-term PIM harms in elderly surgical patients and establish continuous preoperative-intraoperative exposure as a high-risk indicator requiring clinical prioritization.

Moreover, importantly, this study underscores methodological challenges in quantifying cumulative perioperative PIM effects. While preoperative medication use independently predicts prolonged hospitalization and aids risk stratification, regression variance often reflects intraoperative drug characteristics that modify preoperative risk estimates—factors rarely incorporated in existing models [30, 31]. Crucially, we demonstrate that combined preoperative-intraoperative PIMs exposure significantly elevates hospitalization risk beyond single-phase exposure across modeling approaches, suggesting co-exposure drives previously observed preoperative PIM-outcome associations [32, 33]. This supports integrating perioperative factors to optimize predictive models [34, 35]. Notably, comorbidity burden amplifies PIM-related adverse effects by compromising physiological tolerance to drug toxicity, potentially extending hospitalization through intensified monitoring [36, 37]. Consequently, sustained dual-phase PIM exposure warrants personalized deprescribing interventions in vulnerable elderly surgical patients.

### Deprescribing windows and intraoperative optimization

Given this point that elderly patients with perioperative cumulative PIMs exposure face elevated delayed discharge risks after elective non-cardiac surgery, necessitating resource-intensive care. This warrants interventional studies evaluating PIMs deprescribing efficacy [38, 39], which should address intervention feasibility and sustainability trade-offs. Our restricted cubic spline analysis indicates that Pre-PIMs' primary effect occurs during the transition from no exposure to a little/some exposures, consistent with prior evidence that preoperative PIM risks persist perioperatively despite short-term exposure [40, 41]. Thus, the preoperative phase represents both a vulnerability window and strategic opportunity for deprescribing interventions to improve postoperative outcomes。

Conversely, intraoperative PIMs exposure showed only moderate, non-significant association with prolonged hospitalization risk, though the confidence interval's lower limit approached 1—potentially indicating significance with larger samples. This may reflect challenges in translating "potentially inappropriate" to "absolutely inappropriate" medication identification during surgery, where harm-benefit assessments are clinically complex [42]. Nevertheless, clinicians should still rigorously evaluate PIM risks versus benefits in elderly surgical patients, omitting nonessential agents or substituting less toxic alternatives to optimize outcomes and resource utilization. For instance, while postoperative nausea and vomiting prophylaxis remains indicated, geriatric patients—particularly males—rarely require multi-agent regimens given their lower incidence [43, 44]. Similarly, opioid-sparing anesthesia (e.g., ketamine, dexmedetomidine, or lidocaine) demonstrates efficacy in reducing opioid-related adverse effects while maintaining analgesia [45–49]. Collectively, these findings underscore the inherent challenges in perioperative pain management for older adults.

### Clinical practice implications and intervention pathways

This study enhances surgical care quality and value by addressing the unmet need for identifying patients at risk of adverse postoperative outcomes [50]. Pre-PIMs and Intra-PIMs serve as clinically useful predictors for prolonged hospitalization risk, with their combination significantly improving short-term outcome prediction—guiding individualized deprescribing. Optimizing recovery requires: 1) medication reconciliation to identify PIMs, 2) contextual medication assessment minimizing perioperative PIM exposure, and 3) multidisciplinary collaboration including pharmacists to facilitate deprescribing, despite existing workflow barriers to implementing Beers Criteria [51, 52]. However, optimal deprescribing approaches and their risk-benefit profiles remain uncertain [53], and our findings provide preliminary evidence requiring validation through large-scale prospective studies that account for perioperative context and competing risks.

### Limitations and future research

This study has methodological limitations. First, the single-center retrospective design precludes causal inferences and limits generalizability. Second, while unmeasured confounding from undiagnosed conditions could theoretically influence PIM effects, we mitigated this through adjustment for disease severity, functional status, and case complexity. Importantly, medical records cannot establish causality between PIMs and adverse outcomes in comorbid elderly patients—elevated PIM exposure serves as an early risk indicator rather than causative factor. Third, we did not evaluate differential risks across PIM subcategories, precluding drug-specific conclusions. Fourth, as the first Chinese cohort study on perioperative PIM prognosis, sample size restrictions limited subgroup analyses and complication assessments (particularly drug-related events like delirium/falls), potentially underestimating confounding and biasing results away from null. Fifth, residual confounding from unmeasured variables remains possible. Finally, heterogeneity in perioperative practices further constrains generalizability to other settings. Future large-scale prospective studies with granular surgical data are needed to establish causality and identify PIM-specific risks.

## Conclusion

Short-term perioperative PIM prescribing remains highly prevalent among elderly surgical patients. We established a significant dose-response relationship wherein rising cumulative PIMs exposure levels correspond to stepwise increases in prolonged hospitalization risk, with effect magnitudes varying by surgical phase. Crucially, successive preoperative and intraoperative PIMs exposure demonstrated synergistic effects on hospitalization risk—effects further amplified by greater comorbidity burden. Consequently, incorporating dynamic perioperative PIM exposure patterns into prognostic risk stratification may optimize prediction tools for postoperative adverse outcomes. While prospective studies are needed to confirm causal links between transient PIM exposure and surgical outcomes, our findings indicate that enhanced adherence to Beers Criteria recommendations adds substantial value to patient care. This underscores the clinical importance of extending recommendations for PIMs deprescribing to the immediate perioperative setting amid growing institutional and national quality oversight.

## Supplementary Information

Supplementary Material 1.

## Data Availability

The datasets used and analyzed during the current study are available from the corresponding author on reasonable request.
