# Supplementary material for: Association between perioperative potentially inappropriate medication exposure levels and postoperative hospital length of stay among chinese older hospitalized patients: a retrospective cohort study
Source: BMC Geriatr. 2025 Dec 17;26:103. doi: 10.1186/s12877-025-06848-y (PMC12837001; doi:10.1186/s12877-025-06848-y)
Supplement: Supplementary file 1 — Supplementary Material 1. [file 12877_2025_6848_MOESM1_ESM.docx]

**Supplementary table** Baseline characteristics of study population by perioperative cumulative PIMs exposure category

| Characteristics | No exposure，n=61 | Low-moderate exposure, n=210 | High exposure, n=168 | *P* |
| --- | --- | --- | --- | --- |
| Demographics |  |  |  |  |
| Age, years |  |  |  | 0.180 |
| ＜70 | 31 (50.1) | 94 (44.8) | 64 (38.1) |  |
| ≥70 | 30 (49.2) | 116 (55.2) | 104 (61.9) |  |
| Sex |  |  |  | 0.052 |
| Male | 41 (67.2) | 109 (51.9) | 83 (49.4) |  |
| Female | 20 (32.8) | 101 (48.1) | 85 (50.6) |  |
| BMI, kg/m^2^ |  |  |  | 0.160 |
| ＜23 | 22 (36.1) | 87 (41.4) | 82 (48.8) |  |
| ≥23 | 39 (63.9) | 123 (58.6) | 86 (51.1) |  |
| Comorbidities |  |  |  |  |
| Pulmonary circulatory disease | 8 (13.1) | 39 (18.6) | 36 (21.4) | 0.359 |
| Diabetes mellitus | 8 (13.1) | 44 (21.0) | 54 (32.1) | 0.004 |
| Cardiac-cerebral vascular disease | 11 (18.0) | 47 (22.4) | 59 (35.1) | 0.005 |
| Neurodegenerative disease | 1 (1.6) | 9 (4.3) | 25 (14.9) | ＜0.001 |
| Rheumatic disease | 0 (0.0) | 10 (4.8) | 27 (16.1) | ＜0.001 |
| Cancer | 11 (18.3) | 66 (31.4) | 62 (36.9) | 0.025 |
| Peptic ulcer | 6 (9.8) | 15 (7.1) | 14 (8.3) | 0.772 |
| Hypertension | 35 (57.4) | 118 (56.2) | 102 (60.7) | 0.671 |
| CCI |  |  |  | ＜0.001 |
| ＜5 | 42 (68.9) | 99 (47.1) | 58 (34.5) |  |
| ≥5 | 19 (31.1) | 111 (52.9) | 110 (65.5) |  |
| ASA |  |  |  | ＜0.001 |
| ＜3 | 58 (95.1) | 167 (79.5) | 100 (59.5) |  |
| ≥3 | 3 (4.9) | 43 (20.5) | 68 (40.5) |  |
| Site of surgery |  |  |  | ＜0.001 |
| Peripheral | 19 (31.1) | 61 (29.0) | 81 (48.2) |  |
| Intra-thoracic or abdominal | 15 (24.6) | 71 (33.8) | 49 (29.2) |  |
| Pelvic | 27 (44.3) | 78 (37.1) | 38 (22.6) |  |
| Type of anesthesia |  |  |  | ＜0.001 |
| Spinal or epidural | 17 (27.9) | 152 (72.4) | 111 (66.1) |  |
| Nerve block | 8 (13.1) | 15 (7.1) | 17 (10.1) |  |
| General | 36 (59.0) | 43 (20.5) | 40 (23.8) |  |
| Duration of anesthesia, min |  |  |  | ＜0.001 |
| ＜120 | 49 (80.3) | 100 (47.6) | 49 (29.2) |  |
| ≥120 | 12 (19.7) | 110 (52.4) | 119 (70.8) |  |
| Preoperative biochemical values |  |  |  |  |
| Creatinine clearance rate, mL/min |  |  |  | 0.275 |
| ＜60 | 17 (27.9) | 60 (28.6) | 60 (35.7) |  |
| ≥60 | 44 (72.1) | 150 (71.4) | 108 (64.3) |  |
| Albumin, g/L, median (25th, 75th) | 42.0 (39.2, 44.2) | 40.5 (36.1, 44.5) | 35.3 (31.3, 39.8) | ＜0.001 |
| Hemoglobin, g/L, median (25th, 75th) | 132.0 (119.0, 140.0) | 126.0 (111.3, 136.8) | 109.0 (94.8, 123.0) | ＜0.001 |

**Supplementary table** continued

| Characteristics | No exposure，n=61 | Low-moderate exposure, n=210 | High exposure, n=168 | *P* |
| --- | --- | --- | --- | --- |
| Postoperative complication morbidity |  |  |  | ＜0.001 |
| No complication | 61 (100.0) | 186 (88.6) | 122 (72.6) |  |
| Incidence of 1 or more complications | 0 (0.0) | 24 (11.4) | 46 (27.4) |  |

All categories are shown as frequency and (percentage) unless otherwise indicated. Abbreviations: BMI, body mass index; CCI, Charlson Comorbidity Index; ASA, American Society of Anesthesiology; PIM, potentially inappropriate medication.
